# Pulmonary Embolism in Patients Admitted With Takotsubo Cardiomyopathy: Prevalence and Associated In-Hospital Adverse Events

**DOI:** 10.7759/cureus.59268

**Published:** 2024-04-29

**Authors:** Omar Elkattawy, Antonia Sames, Sruthi Kunamneni, Riya Sutariya, Mohamed Ismail, Omar Mohamed, Thomas J Lee, Jahanzeb Javed, Sherif Elkattawy, Afif Hossain, Fayez Shamoon

**Affiliations:** 1 Internal Medicine, Rutgers University New Jersey Medical School, Newark, USA; 2 Medicine, Saint Barnabas Medical Center, Livingston, USA; 3 Cardiology, Saint Joseph's University Medical Center, Paterson, USA

**Keywords:** pulmonary embolism, takotsubo cardiomyopathy, preventive cardiology, cardio-obstetrics, cardiology

## Abstract

Introduction

Takotsubo cardiomyopathy (TCM) is a poorly understood condition typically characterized by abnormal left ventricular wall motion without causative coronary artery disease and typically presents in post-menopausal women after the experience of a physical or emotional stressor. The pathophysiology of TCM is complex and multifactorial, resulting in complications with varied severity; one of the most concerning complications is thromboembolism, specifically, pulmonary embolism (PE), which is understudied in its relation to TCM. The purpose of this study was to characterize and evaluate the real-world prevalence and outcomes of PE in TCM.

Methods

Data were derived from the National Inpatient Sample database from January 2016 to December 2019. The primary outcomes assessed were baseline and hospital admission characteristics and comorbidities for patients with TCM with and without PE. Outcomes for TCM patients with PE and predictors of mortality in TCM were also analyzed.

Results

PE developed in 788 of 40,120 patients with TCM (1.96%). After multivariate adjustment, PE was found to be independently associated with intracardiac thrombus (adjusted odds ratio (aOR) 2.067; 95% confidence interval (CI): 1.198-3.566; p = 0.009) and right heart catheterization (RHC) (aOR: 1.971; 95% CI: 1.160-3.350; p = 0.012). Mortality in patients with TCM was associated with, among other factors, age in years at admission (aOR: 1.104; 95% CI: 1.010-1.017; p = 0.001), African American race (aOR: 1.191; 95% CI: 1.020-1.391; p = 0.027), Asian or Pacific Islander race (aOR: 1.637; 95% CI: 1.283-2.090; p = 0.001), coagulopathy (aOR: 3.393; 95% CI: 2.889-2.986; p = 0.001), liver disease (aOR: 1.446; 95% CI: 1.147-1.824; p = 0.002), atrial fibrillation (aOR: 1.460; 95% CI: 1.320-1.615; p = 0.001), and pulmonary embolism (aOR: 2.217; 95% CI: 1.781-2.760; p = 0.001).

Conclusion

In a large cohort of patients admitted with TCM, we found the prevalence of PE to be 1.96%. PE, along with comorbidities such as coagulopathy and atrial fibrillation, was found to be a significant predictor of mortality in this patient cohort.

## Introduction

Takotsubo cardiomyopathy (TCM) is an understudied syndrome that is typically characterized by abnormal left ventricular wall motion without evidence of attributable coronary artery disease (CAD) [1]. The most commonly described movement is apical ballooning, but other anatomic patterns have also been observed which distinguish subtypes of TCM [1]. Of all patients who present with acute coronary syndrome (ACS), TCM accounts for 1-4% [1]. Nearly 90% of patients diagnosed with TCM are post-menopausal women, and the incidence is highest in Asian and Caucasian populations [1]. Patients classically present with symptoms concerning for ACS, including chest pain, dyspnea, syncope, and palpitations [1]. International diagnostic criteria have been developed to identify TCM cases and distinguish them from acute myocardial infarction, as they both present with ECG abnormalities like ST-segment elevation and/or pronounced T-wave inversions [2,3]. Left ventriculography during catheterization is frequently used to further confirm left ventricular wall motion abnormalities that extend beyond the domain of a single epicardial artery distribution [4]. However, it is important to note that while TCM generally presents in the absence of CAD, patients may also have concomitant CAD, which complicates its diagnosis [1].

Interestingly, the pathophysiology of TCM is disputed [1]. The onset of TCM typically follows the experience of a physical or emotional stressor, suggesting sympathetic nervous system overstimulation and catecholamine release. While some studies have shown higher levels of circulating catecholamines in the subacute phase of TCM compared to myocardial infarction, other studies have failed to demonstrate higher circulating levels during the acute phase [1]. Alternatively, adrenergic receptor function has been postulated to contribute to TCM pathology via a protective switch to β2 receptor signaling in the presence of elevated epinephrine levels [1]. Other proposed mechanisms include endothelial dysfunction, microvascular vasospasm, hormone-mediated changes, genetic predispositions, and pro-inflammatory states [1,3,5]. Angiotensin-converting enzyme (ACE) inhibitors and angiotensin receptor blockers (ARBs) have been shown to improve survival, whereas β-blockers have not been shown to benefit recovery despite frequent use [1]. Anticoagulation can be prescribed for patients at high risk for thromboembolism, and antiplatelet agents such as aspirin can be used, though there is no evidence to suggest cardiovascular benefit [1]. Importantly, no randomized clinical trials have been conducted to date to support treatment recommendations for TCM [5].

In most patients, TCM is transient, and left ventricular ejection fraction (LVEF) is recovered spontaneously [1]. One of the most serious complications of TCM, however, is thromboembolism, which has been reported in 2-14% of patients with TCM [6]. Thromboembolic events occur most commonly in patients with low LVEF (<30%) and detectable apical ballooning and are treated like other thromboembolic diseases with unfractionated heparin, low molecular weight heparin, and vitamin K epoxide reductase antagonists [7]. Pulmonary embolism (PE), a lethal manifestation of post-TCM thromboembolism, is underreported in the literature [8,9]. The first reported case of concomitant TCM and PE was in the setting of COVID-19, which causes a hypercoagulable state and can present with PE [8]. Similarly, the first case of TCM in the setting of PE was reported in a postmenopausal woman with thrombotic disease [9]. Apart from these stand-alone cases, little has been published describing the association between PE and TCM. Thus, this article aims to explore the prevalence, predictors, and in-hospital incidence of PE in patients with TCM.

## Materials and methods

Data acquisition

This is a retrospective study that obtained data from The National Inpatient Sample (NIS) database. The NIS is part of the Healthcare Cost and Utilization Project (HCUP) set forth by the Agency for Healthcare Research and Quality. It utilizes the International Classification of Disease, Tenth Edition, Clinical Modification (ICD-10-CM) codes for diagnosis and procedures. The data set was utilized to examine patients admitted between 2016 and 2019. Encounters with a primary diagnosis of TCM were selected using ICD-10 code I51.81. This cohort of patients was further divided into patients who developed PE versus patients without PE. Adult patients ≥ 18 years old were included. We abstracted data from 40,144 charts, excluded 24, and were left with 40120 charts for analysis. IRB approval was not required as the NIS database provides de-identified patient information.

Outcomes and variables 

Patient baseline characteristics such as age, sex, race, and insurance status were extracted. Comorbidities, hospital complications, mortality rates, disposition status, length of stay, and total charges were also analyzed. 

The primary aim of the study was to assess whether or not there is a difference in outcomes (mortality, in-hospital complications, length of stay, total charges) between the cohort of patients with TCM and PE vs. patients with TCM and without PE. We also analyzed the independent association of PE with outcomes after controlling for confounders such as age, race, sex, and comorbidities. 

Statistical analysis 

Categorical values were analyzed via Pearson's Chi-square analysis and continuous variables were analyzed via independent Student’s t-test. Logistic regression was performed to generate odds ratios (ORs) with 95% confidence intervals (CIs) to assess predictors of mortality in patients with TCM. We also used logistic regression to assess the independent association of PE with clinical outcomes after controlling for confounders like age, sex, race, and comorbidities. A p-value of <0.05 was considered statistically significant. All analyses were completed using IBM SPSS Statistics for Windows, Version 29.0 (Released 2023; IBM Corp., Armonk, New York, United States). 

## Results

This study looked at a total of 40,120 patients admitted with TCM. Of these patients, 788 (1.96%) developed PE. The baseline characteristics of the study population categorized by patients presenting with PE (vs. no PE) in TCM are summarized in Table 1. The age of patients with TCM with PE was slightly lower than the age of patients with TCM without PE, at 65.01 and 67.19 years, respectively (p = 0.001). The length of hospital stay was significantly longer for TCM patients with PE compared to TCM patients without PE (13.96 vs. 6.70 days; p = 0.001). The total mean charges incurred by TCM patients with PE were significantly higher than charges for TCM patients without PE ($196,486.22 vs. $98,333.66; p = 0.001). Patients with PE in TCM were less likely to have routine discharges than those without PE (221 (28.0%) vs. 21020 (53.5%); p = 0.001) and were more likely to die in hospital (121 (15.4%) vs. 2378 (6.0%); p = 0.001). The prevalence of PE was higher in male patients with TCM (213 (27.0%) vs. 6603 (16.8%); p = 0.001). Additionally, the prevalence of PE was higher in African American and Hispanic patients with TCM (94 (12.4%) vs. 3045 (8.0%); p = 0.001 and 56 (7.4%) vs. 2389 (6.3%); p = 0.001, respectively).

**Table 1:** Baseline characteristics of the study population of TCM stratified according to VT status The data has been represented as n and percentage. P-values are significant at <0.05. PE: pulmonary embolism; TCM: takotsubo cardiomyopathy; VT: ventricular tachycardia

Variable	PE	No PE	p-value
Age in years at admission (years)	65.01	67.19	0.001
Gender	-	-	0.001
Male	213(27.0)	6603(16.8)	-
Female	575 (73)	32723 (83.2)	-
Length of stay (days)	13.96	6.70	0.001
Total charges ($)	196,486.22	98,333.66	0.001
Disposition of patient	-	-	0.001
Routine	221 (28.0)	21020 (53.5)	-
Transfer to a short-term hospital	24 (3.0)	1094 (2.8)	-
Transfer other: includes skilled nursing facility (SNF), intermediate care facility (ICF), and another type of facility	271 (34.4)	8263 (21.0)	-
Home health care (HHC)	148 (18.8)	6230 (15.8)	-
Against medical advice (AMA)	3 (0.4)	331 (0.8)	-
Died in hospital	121 (15.4)	2378 (6.0)	-
Primary expected payer	-	-	0.105
Medicare	477 (60.5)	24999 (63.3)	-
Medicaid	100 (12.7)	4083 (10.4)	-
Private insurance	176 (22.3)	8322 (21.2)	-
Self-pay	15 (1.9)	1044 (2.7)	-
No charge	3 (0.4)	70 (0.2)	-
Other	17 (2.2)	771 (2.0)	-
Race	-	-	0.001
White	558 (73.5)	30757 (80.8)	-
Black	94 (12.4)	3045 (8.0)	-
Hispanic	56 (7.4)	2389 (6.3)	-
Asian or Pacific Islander	7 (0.9)	784 (2.1)	-
Native American	4 (0.5)	248 (0.7)	-
Other	40 (5.3)	848 (2.2)	-

Univariate analysis results showing the associations between several comorbidities and PE in TCM are depicted in Table 2. Patients who developed PE had more comorbidities including iron deficiency anemia (55 (7.0%) vs. 1666 (4.2%); p = 0.001), coagulopathy (64 (8.1%) vs. 1140 (2.9%); p = 0.001), liver disease (35 (4.4%) vs. 935 (2.4%); p = 0.001), pulmonary hypertension (77 (9.8%) vs. 2636 (6.7%); p = 0.001), opioid use (36 (4.6%) vs. 1227 (3.1%); p = 0.021), and obesity (109 (13.8%) vs. 4508 (11.5%); p = 0.039). Patients without PE had a higher burden of type 2 diabetes mellitus (T2DM) (8954 (22.8%) vs. 133 (16.9%), p=0.001), hypertension (14303 (36.4%) vs. 206 (26.1%), p=0.001), peripheral vascular disease (945 (2.4%) vs. 10 (1.3%), p=0.039), hypothyroidism (6987 (17.8%) vs. 107 (13.6%), p=0.002), coronary artery disease (15237 (38.7%) vs. 192 (24.4%), p=0.001), and tobacco use disorder (400 (1%) vs. 2 (0.3%), p=0.033). 

**Table 2:** Prevalence of comorbidities in the study population of TCM patients with and without PE The data has been represented as n (%). p-values are significant at <0.05. PE: pulmonary embolism; COPD: chronic obstructive pulmonary disease; IDA: iron deficiency anemia; T2DM: type 2 diabetes mellitus; HTN: hypertension; PVD: peripheral vascular disease; AF: atrial fibrillation; HIV: human immunodeficiency virus; CAD: coronary artery disease; TUD: tobacco use disorder; OSA: obstructive sleep apnea; CUD: cocaine use disorder; OUD: opioid use disorder; ESRD: end-stage renal disease; TCM: takotsubo cardiomyopathy

Variable	PE	No PE	p-value
COPD	229 (29.1)	13217 (33.6)	0.007
IDA	55 (7.0)	1666 (4.2)	0.001
Coagulopathy	64 (8.1)	1140 (2.9)	0.001
Cerebrovascular disease	15 (1.9)	576 (1.5)	0.311
T2DM	133 (16.9)	8954 (22.8)	0.001
HTN	206 (26.1)	14303 (36.4)	0.001
Alcohol abuse	42 (5.3)	2290 (5.8)	0.559
Liver disease	35 (4.4)	935 (2.4)	0.001
PVD	10 (1.3)	945 (2.4)	0.039
AF	170 (21.6)	8099 (20.6)	0.5
Hypothyroidism	107 (13.6)	6987 (17.8)	0.002
HIV	3 (0.4)	86 (0.2)	0.338
CAD	192 (24.4)	15237 (38.7)	0.001
Pulmonary HTN	77 (9.8)	2636 (6.7)	0.001
TUD	2 (0.3)	400 (1.0)	0.033
OSA	49 (6.2)	2239 (5.7)	0.529
CUD	10 (1.3)	321 (0.8)	0.164
OUD	36 (4.6)	1227 (3.1)	0.021
Obesity	109 (13.8)	4508 (11.5)	0.039
ESRD	19 (2.4)	799 (2.0)	0.455

A summary of crude analysis results of clinical outcomes of TCM patients with and without PE is summarized in Table 3. Patients with PE had higher rates of cardiac arrest (38 (4.8%) vs. 1235 (3.1%); p=0.008), intracardiac thrombus (16 (2.0%) vs. 291 (0.7%); p=0.001), cardiogenic shock (79 (10.0%) vs. 2581 (6.6%); p=0.001), vasopressor use (31 (3.9%) vs. 898 (2.3%), p=0.002), and right heart catheterizations (17 (2.2%) vs. 283 (0.7%), p=0.001). Non-ST elevation myocardial infarction (NSTEMI) was less frequently seen in PE patients (143 (18.1%) vs. 9292 (23.6%); p=0.001). Compared to patients with PE, those who did not develop PE were more likely to have permanent pacemakers placed (4085 (10.4%) vs. 50 (6.3%); p=0.001), undergo left heart catheterization (16305 (41.5%) vs. 165 (20.9%); p=0.001), angioplasty (1015 (2.6%) vs. 10 (1.3%); p=0.021), and had higher rates of NSTEMIs (9292 (23.6%) vs. 143 (18.1%); p=0.001). 

**Table 3:** Outcomes for the study population of TCM patients with and without PE The data has been represented as n (%). p-values are significant at <0.05. PE: pulmonary embolism; PPM: permanent pacemaker; VF: ventricular fibrillation; VT: ventricular tachycardia; RHC: right heart catheterization; LHC: left heart catheterization; IABP: intra-aortic balloon pump; STEMI: ST-segment elevation myocardial infarction; NSTEMI: non-ST elevation myocardial infarction; TCM: takotsubo cardiomyopathy

Variable	PE	No PE	p-value
Cardiac arrest	38 (4.8)	1235(3.1)	0.008
PPM	50 (6.3)	4085 (10.4)	0.001
VF	16 (2.0)	574 (1.5)	0.187
VT	49 (6.2)	1876 (4.8)	0.060
Angioplasty	10 (1.3)	1015 (2.6)	0.021
Intracardiac thrombus	16 (2.0)	291 (0.7)	0.001
RHC	17 (2.2)	283 (0.7)	0.001
LHC	165 (20.9)	16305 (41.5)	0.001
Cardiogenic shock	79 (10.0)	2581 (6.6)	0.001
IABP	11 (1.4)	554 (1.4)	0.976
Mechanical ventilation	52 (6.6)	2200 (5.6)	0.225
Vasopressor use	31 (3.9)	898 (2.3)	0.002
STEMI	17 (2.2)	1264 (3.2)	0.095
NSTEMI	143 (18.1)	9292 (23.6)	0.001

We used multivariate logistic regression to assess the independent association of pulmonary embolism with outcomes in TCM patients, controlling for confounding variables such as sex, race, age, and comorbidities. After adjustment, PE was independently associated with increased frequency of intracardiac thrombus (adjusted odds ratio (aOR): 2.067; 95% CI: 1.198-3.566; p = 0.009) and right heart catheterization (aOR: 1.971; 95% CI: 1.160-3.350; p = 0.012) compared to patients without PE. PE in TCM was independently associated with less frequent left heart catheterization (aOR: 0.524; 95% CI: 0.433-0.634; p = 0.001) compared to patients without PE.

**Figure 1:**
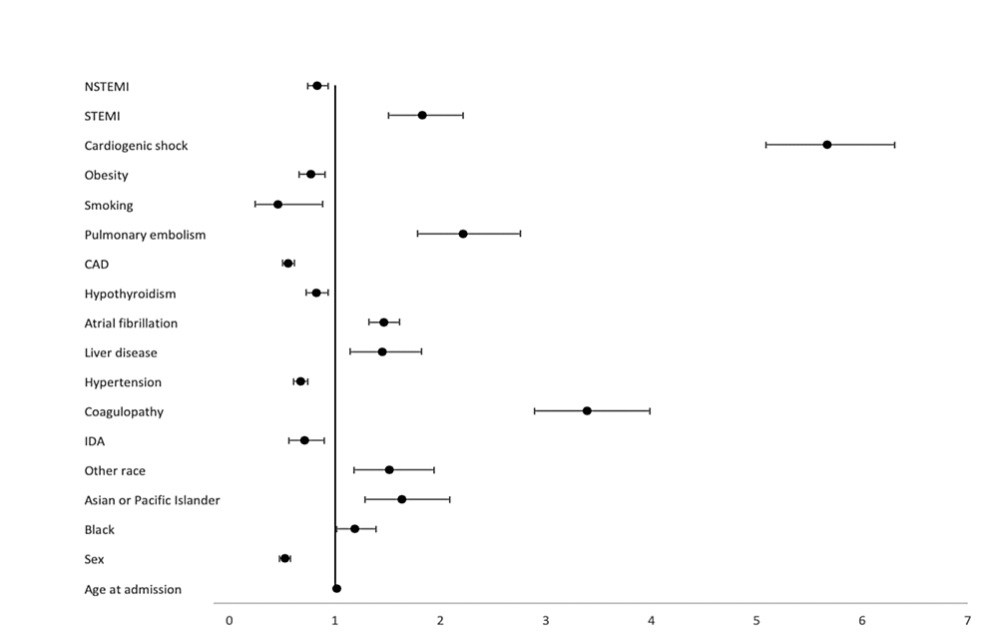
Predictors of mortality in TCM patients NSTEMI: non-ST-elevation myocardial infarction; STEMI: ST-elevation myocardial infarction; CAD: coronary artery disease; IDA: iron deficiency anemia; TCM: takotsubo cardiomyopathy

We conducted a second multivariate logistic regression to evaluate predictors of mortality in TCM patients (Figure 1). Variables that were significantly associated with increased risk of mortality included age in years at admission (aOR: 1.104; 95% CI: 1.00-1.017; p = 0.001), African American race (aOR: 1.191; 95% CI: 1.020-1.391; p = 0.027), Asian or Pacific Islander race (aOR: 1.637; 95% CI: 1.283-2.090; p = 0.001), and other race (aOR: 1.514; 95% CI: 1.181-1.941; p = 0.001). We also found that mortality in patients with TCM was associated with coagulopathy (aOR: 3.393; 95% CI: 2.889-2.986; p = 0.001), liver disease (aOR: 1.446; 95% CI: 1.147-1.824; p = 0.002), atrial fibrillation (OR: 1.460; 95% CI: 1.320-1.615; p = 0.001), pulmonary embolism (aOR: 2.217; 95% CI: 1.781-2.760; p = 0.001), cardiogenic shock (aOR: 5.663; 95% CI: 5.085-6.506; p = 0.001), and STEMI (aOR: 1.829; 95% CI: 1.508-2.218; p = 0.001). Interestingly, survival in patients with TCM was associated with the female sex (aOR: 0.523; 95% CI: 0.472- 0.579; p=0.001), iron-deficiency anemia (aOR: 0.712; 95% CI: 0.563-0.901; p = 0.005), hypertension (OR: 0.673; 95% CI: 0.608-0.744; p = 0.001), hypothyroidism (aOR: 0.827; 95% CI: 0.728-0.939; p = 0.003), coronary artery disease (aOR: 0.558; 95% CI: 0.505-0.617; p = 0.001), smoking (aOR: 0.462; 95% CI: 0.242-0.880; p = 0.019), obesity (aOR: 0.774; 95% CI: 0.661-0.907; p = 0.002), and NSTEMI (aOR: 0.833; 95% CI: 0.745-0.932; p = 0.001).

## Discussion

In the 40,120 patients with TCM studied, the prevalence of PE was 1.96%. Patients with TCM presenting with PE had longer hospital stays, higher total charges, and higher mortality rates. In the cohort of TCM patients studied, a higher incidence of PE was found in males. Previous literature notes that despite higher rates of TCM in females, males are more likely to have adverse clinical outcomes when presenting with TCM [10]. A variety of findings including an exaggerated inflammatory response, relatively lower levels of estrogen, which normally provides a cardioprotective effect, and a difference in the type of stressors in males compared to females have been suggested as possible explanations, although the underlying mechanisms for the difference in the severity of clinical outcomes remain to be explored [11,12].

Additionally, in the cohort studied, African American and Hispanic patients were more likely to present with PE. Previous studies have shown a greater prevalence of PE in African American patients without TCM in comparison to White patients [13,14], although the same has not been found for Hispanic patients [13,15]. Currently, evidence suggests that racial disparities in venous thromboembolism between African American and White patients are due to greater risk factors such as higher body weight and factor VIII concentrations, as well as lower family income [16]. However, limited literature is published regarding Hispanic patients and TCM, and further research is needed to understand the higher incidence of PE in Hispanic patients with TCM.

Patients with PE and TCM were found to have a higher burden of iron-deficiency anemia. Recent case reports have noted an association between iron-deficiency anemia and thromboembolism [17,18]. While the relationship between iron-deficiency anemia and thromboembolism is not completely understood, the most probable mechanism is that iron-deficiency anemia results in thrombocytosis via decreased inhibition of thrombopoiesis, which leads to an increased risk of thromboembolism [19]. This association may also explain why TCM patients with iron-deficiency anemia have lower odds of mortality; the easily treatable nature of iron-deficiency anemia facilitates the decrease of risk factors for coagulopathy and thus mortality [18]. 

We also observed that smoking, obesity, coronary artery disease, hypertension, and hyperthyroidism decreased the odds of mortality in TCM patients. It seems contradictory that conditions such as smoking and obesity, which are known to contribute to the pathogenesis of cardiovascular disease, were shown to decrease the odds of mortality. The potential advantage of excess adipose storage during illness is an often-cited explanation [20]. However, a recent investigation found that the “obesity paradox” is a product of reverse causation and confounding by smoking; since individuals with obesity are less likely to smoke than individuals of normal weight and are selected into a disease group for which both smoking and obesity are risk factors, selection bias and downward bias may occur, leading to statistical error [20]. 

The contributions of hypertension and coronary artery disease to decreased mortality in TCM patients are also perplexing. To our knowledge, there is no literature suggesting that hypertension or coronary artery disease decreases mortality for TCM. It is possible that therapies targeting pre-existing hypertension and coronary artery disease in TCM patients improve outcomes in TCM patients, though further studies should be conducted to evaluate this relationship further.

Finally, we found that hyperthyroidism decreases the odds of mortality in TCM patients. A recent meta-analysis found that both overt and subclinical hyperthyroidism are associated with an increased risk of cardiovascular events and cardiovascular mortality [21]. Common cardiovascular symptoms in hyperthyroid patients include palpitation, atrial fibrillation, and various arrhythmias [22]. Perhaps because of, not despite, this close relationship with cardiovascular function, the management of hyperthyroidism via diuretics and β-blockers may decrease the risk for mortality in TCM patients. Most importantly, hyperthyroidism increases the density of β1-adrenergic receptors on the myocardium [22], which increases sensitivity to circulating catecholamines and counteracts the upregulation of β2 receptors thought to contribute to the pathogenesis of TCM [1,23].

Limitations of our study include that it is observational, therefore our results merely reflect correlation, and caution must be exercised when drawing conclusions. Because the NIS is an administrative database that uses ICD-10 codes, it is prone to human errors in coding. Other confounders may be present that are not controlled for in our study, including medication use that is not provided by the NIS. Results from our study can only be interpreted in the inpatient setting and cannot extend to outpatient practice. Patients are also not followed after discharge; therefore, long-term outcomes for patients cannot be established. We did not study the treatment modality of PE in our patients; therefore, outcomes may differ depending on which treatment (anticoagulation vs. lysis vs. endovascular therapy) the patient received. Further studies could prospectively study the impact of the different modalities of PE treatment on outcomes among patients hospitalized with TCM.

## Conclusions

In this large real-world registry of patients with TCM, we found the prevalence of PE in patients with TCM to be 1.96%. PE in patients with TCM was found to be independently associated with increased outcomes of intracardiac thrombus and right heart catheterization. Mortality in patients with TCM was associated with age in years at admission, race, and various cardiac and pulmonary complications including PE.
